# The Role of Motivation Systems, Anxiety, and Low Self-Control in Smartphone Addiction among Smartphone-Based Social Networking Service (SNS) Users

**DOI:** 10.3390/ijerph19116918

**Published:** 2022-06-05

**Authors:** Min-Jung Kwak, Hyun Cho, Dai-Jin Kim

**Affiliations:** 1 Department of Neurobiology, College of Biomedicine and Health Science, Seoul St. Mary’s Hospital, The Catholic University of Korea, Seoul 06591, Korea; watersho1218@gmail.com; 2 Department of Psychiatry, Seoul St. Mary’s Hospital, Seoul 06591, Korea; sonap1@hanmail.net; 3 Department of Psychiatry, College of Medicine, Seoul St. Mary’s Hospital, The Catholic University of Korea, Seoul 06591, Korea

**Keywords:** smartphone addiction proneness, smartphone-based social networking service users, behavioral motivation system, behavioral activation system, self-control, anxiety, path analysis

## Abstract

Given that Social Networking Service (SNS) has emerged as the most influential platform, which can lead users to addictive smartphone use, it is necessary to investigate which psychological variables lead smartphone-based SNS users to addictive smartphone use. Still, studies on the relationship between psychological variables and addictive smartphone use among smartphone-based SNS users remain to be explored. Therefore, this study aims to investigate the role of psychological factors on smartphone addiction proneness (SAP). A total of 433 smartphone-based SNS users were collected from 5003 adults in Korea. Data were analyzed with descriptive statistics, Pearson’s correlation coefficients, and path analysis using SPSS 21.0 and AMOS 23.0. The results of a parallel-mediation path analysis demonstrated that Behavioral Inhibition (BIS), Behavioral activation (BAS) drive, anxiety, and low self-control directly influenced SAP, separately. BIS and BAS _drive also had significant indirect effects on SAP through the effect of anxiety. BIS and BAS_fun had significant indirect effects on SAP through the effect of low self-control. The study variables accounted for 38.4 of the total variances of SAP. Thus, when establishing interventions to reduce the users’ addictive smartphone use, these interactive relationships of the variables should be considered.

## 1. Introduction

It is no exaggeration to mention that the present time is the ‘smartphone era’ [1]. Smartphones are advanced technology devices, which allow users to access beneficial contents and various applications at our fingertips [2]. As smartphones have emerged as a major way of communicating, learning, and entertaining [3], smartphones have become indispensable in our lives. The appropriate use of smartphones benefits users by offering various learning, communicating, and entertaining opportunities regardless of time and place [4]. However, in conjunction with the rapid growth of smartphone users, heavier engagement with smartphones, known as ‘smartphone addiction,’ has emerged as a significant social problem, which negatively impacts the physical, psychological, or social aspects of life [5]. A recent review article on addictive smartphone usage has documented that the prevalence of smartphone addiction rate was ranged from 10% to 30% [6]. Though an official clinical diagnosis does not yet exist in the 5th Edition of Diagnostic and Statistical Manual of Mental Disorders (DSM-5), smartphone addiction has been regarded as a behavioral addiction that mirrors addiction symptomatology, including excessive dependence, obsessive use, withdrawal, and irresistible use despite undesirable outcomes [4]. However, it is still controversial whether it is appropriate to apply the word “addiction” to problematic smartphone use [5]. Therefore, the present study will use alternative terms such as ‘addictive use,’ ‘addiction proneness,’ and ‘problematic use’ for heavy dependence on smartphones in addition to using the term ‘smartphone addiction.’

While research on addictive smartphone use has been prolific, it has been argued that smartphone users do not become addicted to the device itself but to the use of specific applications [4,7,8]. Recent studies have indicated some application contents are more likely to have a significant impact on addictive smartphone use than others [7,9]. That is, similar to alcoholics who are not addicted to a bottle but to drinks inside of the bottle [10], heavy smartphone users are also not addicted to their smartphone devices but the media properties of applications that they use. Among the numerous content types of smartphone use, Social Networking Service (SNS) has emerged as the most influential platform, which can lead users to addictive smartphone use [4,7,11,12]. SNS is a relationship-oriented application such as Facebook, Instagram, Twitter, or WeChat, which enable users to create a personal profile, update their status, and sharing content to manage their social relationships [10]. In recent years, SNS has become an indivisible part of our daily lives with the advent of smartphones, which enable users to use SNS applications anytime and anywhere [12]. Smartphone users mostly use their device for the purpose of using SNS applications [10], and a median of 91% of smartphone users in developing countries tend to use SNS applications [13]. Indeed, the frequent usage of SNS via smartphone can increase addictive smartphone usage behavior. According to a study on investigating what type of content smartphone users are addicted to, SNS was the most powerful application that causes addictive smartphone use [7]. Some foundational studies also reported that those who spent their smartphone usage time mostly on using SNS showed higher smartphone addiction propensity [8,14].

Given that not all smartphone users who mostly use their devices for using SNS show addictive smartphone usage patterns [9], it is necessary to investigate psychological factors and its relationship that may explain the users’ addictive smartphone use. Although several studies have explored several psychological variables on addictive smartphone use considering the SNS usage, e.g., [15,16], the participants in the research were ‘general users’, whose main smartphone usage content has not been explored. Moreover, some studies included the use of SNS as a predictor itself of heavy smartphone use and did not considered SNS usage as a characteristic of participants, e.g., [17]. Additionally, as SNS applications are particularly focused on connecting and socializing people, whereas social media applications refer to the web environment for producing, sharing, and collaborating on content online [10], social media and SNS should be distinguished from each other [10]. However, those two terms have been interchangeably used in previous literature. Thus, exploring the relationship between psychological variables and addictive smartphone use in the sample of smartphone users whose main usage is SNS remains to be more fully explored. 

Therefore, we aim to investigate the role of psychological variables on addictive device use of smartphone users whose main device usage is SNS. The participants in this study will be defined as ‘smartphone-based SNS users.’ Findings from our research will make a unique contribution to this field of research as considering the context of smartphone-based SNS use. 

## 2. Theoretical Background

The existing literature on addictive smartphone and SNS use have highlighted neuropsychological motivation system, such as Behavioral Inhibition System (BIS) and the Behavioral Activation System (BAS) [5], as well as several psychological factors such as anxiety [18,19] and self-control [20,21]. 

The BIS and BAS are basic neuropsychological motivation systems that control behavior and affect [22]. The BIS is responsible for inhibitory behaviors in response to negative affections, such as punishments [23]. The BAS is responsible for activation behaviors in response to positive affections, such as rewards [23]. The BAS consists of three subcomponents: (1) BAS_drive (the persistent pursuit of desired goals); (2) BAS_fun seeking (a desire for novel rewards and a willingness to approach pleasurable events); (3) BAS_reward responsiveness (positive responses to the occurrence of rewards) [22]. 

High engagement in specific behaviors can vary depending on one’s level of the activated motivation associated with negative/positive stimuli [24]. Not surprisingly, it has been suggested that BIS/BAS sensitivity is associated with pathological behavioral engagement, which is addiction [25,26]. Indeed, BIS/BAS have been known to increase one’s vulnerability to addictive behaviors towards substances [27], pathological gambling [28,29], and the Internet [24]. In terms of smartphone addiction, a growing body of literature reported the high BIS and/or BAS sensitivity predispose to high engagement in smartphone and mobile use. According to a study on exploring personality factors predicting smartphone addiction, addictive participants had a higher level of BIS/BAS scores than participants in control group [5]. In other studies, BAS significantly predicted 47% of mobile addiction [24] and mediated the relationship between psychiatric comorbidity and problematic smartphone use [25]. As numerous studies also suggested the individual differences in underlying motives for using SNS, indicating the possible relationship among BIS/BAS and SNS usage, e.g., [26,27], BIS/BAS can be considered as potential variables, which may predict smartphone addiction in the sample of smartphone-based SNS users.

Still, BIS/BAS itself may not sufficiently explain the mechanism of smartphone addiction [5]. Thus, it is necessary to explore the potential psychological mediators, which can bridge the relationship between BIS/BAS and addictive smartphone use. Hence, this study will review a research question that BIS/BAS may affect addictive smartphone use of smartphone-based SNS users while being mediated by other psychological variables. 

Anxiety has been known as a factor that causes the addictive use of smartphone as a consequence of alleviating the negative emotions [18,19]. According to a systematic review of the relationship between smartphone use and psychiatric comorbidities, addictive smartphone usage co-occurred with anxiety [30]. A research on the association between problematic smartphone use and mental health symptoms has shown that high anxiety was positively correlated with habitual smartphone checking behavior, which can lead to increased addictive smartphone use [31]. Since the level of anxiety is also positively related to the use of SNS [8,10,21,32,33,34], anxiety can be considered as a potential predictor of addictive smartphone use in the sample of smartphone-based SNS users. 

It has been suggested that the activation of BIS/BAS accompanies anxiety by manifesting one’s sensitivity to aversive results or non-punishment, e.g., [35,36]. BIS is related to withdrawal behaviors that could cause punishment and one’s motivation to avoid potential threats [37]. Individuals with high BIS, who have high punishment expectancies, will expect negative consequences from their behaviors and experience punishment-related negative emotion such as anxiety [35]. BAS is associates with signals of reward and terminating punishing stimuli [37]. This reward-approaching motivation can activate passive avoidance, characterized as the inhibition of appetite behavior to bring desired consequences in response to punishing cues; in turn, it will produce anxiety [38]. Therefore, the current research will review a research question that anxiety may influence smartphone addiction while mediating the relationship between BIS/BAS and addictive smartphone use, in the sample of smartphone-based SNS users.

Self-control refers to a capacity to consciously exert control over impulses by suppressing, monitoring, and restraining one’s automatic actions, thoughts, and emotions [39]. As the main function of self-control is resisting temptations and inhibiting undesirable behaviors to achieve a primary goal, the failure of self-control is associated with addictive behaviors [40]. Among numerous types of behavioral addiction, previous study results have found that the low level of self-control increased the possibility of addictive smartphone use e.g., [20,39,41,42]. Findings from another study indicated that the low level of self-control was the only factor that was significantly correlated with higher smartphone screen time, and participants with lower self-control tended to have greater difficulty in putting their phones aside than those who have higher self-control [43]. As a lack of self-control is also positively associated with the problematic use of SNS, e.g., [10,14,41,43] the low level of self-control can be considered as a potential factor, which may predict smartphone addiction in the sample of smartphone-based SNS users.

Self-control also associated with behavioral approach and inhibition [44]. Within the motivational framework, BIS/BAS activate sustaining or continuing behaviors when a potential threat or a reward is detected [45]. Regarding these mechanisms, previous research have suggested that the increased level of BIS/BAS is associated with a failure of controlling one’s behaviors [45,46]. As BIS can manifest one’s sensitivity to potential threats, the high level of BIS is related to the activation of not only halting ongoing behavior but also engaging in subsequent reward-seeking behaviors as a part of active avoidance [45]. This mechanism can result in lowering one’s self-control capacity and increasing the likelihood of indulging behaviors [47]. Individuals with high BAS sensitivity tend to have high reward expectancies, anticipate positive consequences from their behaviors, and engage in addictive behaviors that may impair self-control capacity [37]. Considering the theoretical suggestions on a potential association between BIS/BAS and self-control, our study will review a research question that the level of self-control may influence the addictive smartphone use of smartphone-based SNS users while mediating the relationship between BIS/BAS and smartphone addiction. 

## 3. Conceptual Framework

**Figure 1:**
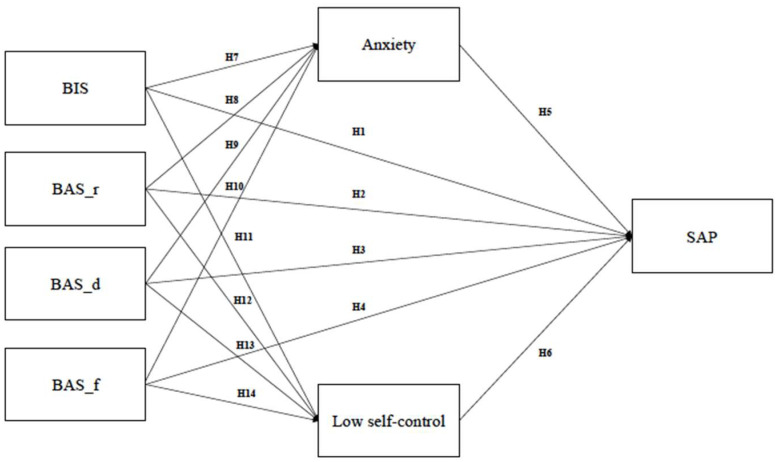
Hypothetical path model diagram. BIS = behavioral inhibition system; BAS_r = behavioral activation system_reward responsiveness; BAS_d = behavioral activation system_drive; BAS_f = behavioral activation system _fun seeking; SAP = Smartphone Addiction Proneness.

The current study aims to investigate the influences of BIS, BAS, anxiety and low self-control on smartphone addiction of smartphone-based SNS users. We conducted the path analysis with the maximum-likelihood estimation to assess the contribution of BIS/BAS, anxiety, low self-control to SAP. Path analysis is a straightforward extension of multiple regression modeling, which can be used to estimate the magnitude and significance of direct and indirect effects, including mediational links [48]. We included BIS, BAS components (BAS_reward responsiveness; BAS_drive; BAS_fun seeking, respectively), anxiety, and low self-control in our hypothesized model of SAP. Considering that the use of SNS applications can affect addictive smartphone use [14], we included the hours of daily SNS applications via smartphones as a control variable for SAP to control the confounding effect of the SNS usage time on SAP. The hypothetical path analysis model of smartphone-based SNS users’ smartphone addiction proneness (SAP) was suggested in Figure 1.

14 hypotheses are proposed as follows:

**H1.**  *BIS has a significant direct effect on SAP*.

**H2.**  *BAS_reward responsiveness has a significant direct effect on SAP.*

**H3.**  *BAS_drive has a significant direct effect on SAP*.

**H4.**  *BAS_fun seeking has a significant direct effect on SAP*.

**H5.**  *anxiety has a significant direct effect on SAP*.

**H6.**  *low self-control has a significant direct effect on SAP*.

**H7.**  *BIS has a significant direct effect on anxiety*.

**H8.**  *BAS_reward responsiveness has a significant direct effect on anxiety*.

**H9.**  *BAS_drive has a significant direct effect on anxiety*.

**H10.**  *BAS_fun seeking has a significant direct effect on anxiety*.

**H11.**  *BIS has a significant direct effect on low self-control*.

**H12.**  *BAS_reward responsiveness has a significant direct effect on low self-control*.

**H13.**  *BAS_drive has a significant direct effect on low self-control*.

**H14.**  *BAS_fun seeking has a significant direct effect on low self-control*.

## 4. Materials and Methods

### 4.1. Participants

A total of 433 participants, whose main smartphone usage was SNS, were chosen among 5003 survey respondents, who were aged 20–49 from metropolitan areas in Korea, after treating missing value and ruling out those with psychiatric disorders. They were offered a web-based consent statement prior to participation. Those who agreed with the consent completed the entire questionnaire with their smartphones. The participants who refused to provide consent were excluded. Original data were collected using an online survey conducted by a professional polling company (Hankook Research, Inc., Seoul, Korea). The selected 433 participants are defined as ‘smartphone-based SNS users’ in this study.

### 4.2. Measures

The survey consisted of the following questionnaires: Demographic information including age, gender, geographical location, educational level, marital status, and socio-economic status, average smartphone usage hours, K-SAPS, BIS/BAS, Brief Self-Control Scale (BSCS), and anxiety subscale from the Symptom Checklist-90-Revised (SCL-90-R). The Korean version of SAPS, BIS/BAS, Brief Self-Control Scale (BSCS), and anxiety subscale from the Symptom Checklist-90-Revised (SCL-90-R) have been checked the validity and reliability validation in the previous studies, e.g., [49,50,51,52]. All questionnaires were self-administered.

#### 4.2.1. Smartphone Addiction Proneness Scale (K-SAPS)

The K-SAPS for adults [48], was used to examine smartphone addiction proneness. The K-SAPS consists of 15 Likert-type items (1 = *Strongly disagree* to 4 = *Strongly agree*). The scale contains four subdomains: (1) disturbance of adaptive functions; (2) virtual life orientation; (3) withdrawal; and (4) tolerance. The sum of all subdomain scores were used to measure smartphone addiction proneness. The cut-off score for the risk of smartphone addiction was 40. In this sample, Cronbach’s alpha was 0.903.

#### 4.2.2. Behavioral Inhibition System (BIS) and Behavioral Activation System (BAS) Scales

The Korean version of the BIS/BAS scales [50] were used. The behavioral inhibition system (BIS) scale [22] is a 7-item self-report questionnaire designed to assess aversive motivation, which is associated with the inhibition of behavior towards goals. Items were rated on a 4-point Likert scale (1 = *Strongly disagree* to 4 = *Strongly agree*). In this sample, Cronbach’s alpha was 0.78. The behavioral activation system (BAS) [22] was used to measure the level of BAS sensitivity associated with the activation of behavior towards goals. The BAS is a 13-item self-report questionnaire designed to assess appetitive motivation in three domains: reward responsiveness, drive and fun seeking. Items were rated on a 4-point Likert scale (1 = *Strongly disagree* to 4 = *Strongly agree*). To examine the unique contribution of subscales for the accuracy of results, the subscales of the BAS were administered in the statistical analysis as subcomponents. In this sample, Cronbach’s alphas were 0.844, 0.769, and 0.772 for reward responsiveness, drive, and fun seeking, respectively. 

#### 4.2.3. The Brief Self-Control Scale

The Korean Version of the Brief Self-Control Scale (BSCS) [51] was used to measure a lack of self-control. The BSCS is a 13-item self-report questionnaire designed to assess one’s ability to control thoughts, emotions, performance regulation, habit breaking, and impulsivity [51]. The items were rated on a 5-point Likert scale (1 = *Not at all* to 5 = *Very much*). Higher scores represent lower levels of self-control. In this sample, Cronbach’s alpha was 0.724.

#### 4.2.4. Anxiety from the Symptom Checklist-90-Revised (SCL-90-R)

The anxiety subscale from the Korean version of SCL-90-R [52] was used in this study. The 10 items were rated on a 5-point Likert scale (0 = *Not at all* to 5 = *Extremely*), indicating the severity of participants’ anxiety symptoms in the past week. In this sample, Cronbach’s alpha was 0.944.

## 5. Statistical Analysis 

All statistical analyses were conducted using SPSS/WIN 21.0 (IBM, Armonk, NY, USA) and AMOS 23.0 (SPSS, Chicago, IL, USA). Descriptive statistics included frequency, mean, standard deviation, and percentage, which were used to describe participants’ sociodemographic characteristics, daily hours of using smartphone and SNS applications, and study variables. Skewness and kurtosis were examined to confirm that study variables were normally distributed.

To evaluate the fitness of the hypothetical path model, the maximum-likelihood estimation method was used. Indicators for evaluating model goodness of fit of the hypothetical path model were the χ^2^ statistic, Root Mean Square Error of Approximation (RMSEA), Comparative Fit Index (CFI), Tucker-Lewis Index (TLI), and Standardized Root Mean Residual (SRMR) were used. The overall fitting model was considered to be acceptable as following values: the RMSEA was 0.10 or lower [53]; the CFI and TLI were all 0.90 or greater and the SRMR was 0.08 or lower [54]. The fitness of the path coefficient the direct and indirect effects of variables related to smartphone addiction proneness were estimated. We used bootstrapping procedure to identify the significant effect of the indirect effects. The number of bootstrapping samples was 5000. The indirect effects which did not include zero in the 95% bootstrap confidence interval proves that the indirect effects are significant and the mediating effects existed [55].

## 6. Results

### 6.1. Participants’ Sociodemographic Characteristics 

Characteristics of participants’ sociodemographic were suggested in Table 1. Of the 433 participants, 218 were male (50.3%) and 215 were female (49.7%). Participants’ age ranged from 20 to 40, and their mean age was 30.75 (SD = 7.84). Most participants graduated university or community college (91.7%), were salaried workers (53.7%), not married (61.7%), and perceived their economic status as low (41.8%) and middle (46.4%).

### 6.2. Smartphone and SNS Application Use among Participants

The daily usage hours of smartphone and SNS applications were depicted in Table 2. The mean duration of daily smartphone use was 10.44 h (SD = 7.73); 33.3% of the participants used their smartphones for 10 h or more. The mean of daily SNS application use was 4.61 (SD = 4.06); 26.1% of the subjects used SNS via their smartphones for 5 h or more. 20.6% of the participants used SNS via their smartphones for 2 h or more once they started using the applications.

### 6.3. Descriptive Statistics and Inter-Correlation Coefficient in Measured Variables

Table 3 summarizes the correlation matrix among the measured variables and the descriptive statistics. The result revealed that the inter-correlations among all variables were significant. Smartphone addiction proneness was positively correlated with all study variables.

The skewness and kurtosis values of all variables did not exceed the absolute value of two. As the correlations among all independent variables did not exceed the criterion value of 0.80 [52], we ruled out the problem of multicollinearity.

### 6.4. Model Fit

In this study, the fitness index was analyzed as χ^2^ = 10.044 (*p* = 0.123), df = 6, CFI = 0.997, TLI = 0.986, RMSEA = 0.039, and SRMR = 0.0426. The statistical value of χ^2^ is inversely proportional to *p*-value; thus, this model can be said to be fit to the data as the null hypothesis was rejected. The overall fit is interpreted as conforming to the acceptance criteria. 

### 6.5. Parameter Estimation and Significance of the Path Model 

**Figure 2:**
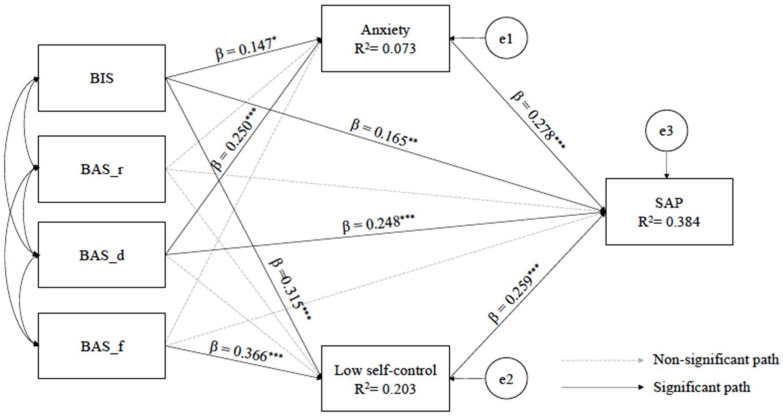
A path diagram of the study. Model fit statistics: χ^2^ = 10.004, df = 6. Solid lines represent significant standardized path coefficients (* *p* < 0.05, ** *p* < 0.01, *** *p* < 0.001). Error variances appear in small circles. BIS = behavioral inhibition system; BAS_r = behavioral activation system_reward responsiveness; BAS_d = behavioral activation system_drive; BAS_f = behavioral activation system _fun seeking; SAP = Smartphone Addiction Proneness.

Figure 2 shows the path based on the parameters of the standardized path of the path model of our study. The paths that had a significant direct effect on anxiety were BIS (β = 0.147, *p* < 0.05) and BAS-drive (β = 0.250, *p* = < 0.001). The paths that had a significant direct effect on low self-control were BIS (β = 0.315, *p* < 0.001) and BAS_fun seeking (β = 0.366, *p* < 0.001). The paths that had a significant direct effect on SAP were BIS (β = 0.165, *p* < 0.01), BAS_drive (β = 0.248, *p* < 0.001), anxiety (β = 278, *p* < 0.001), and low self-control (β = 259, *p* < 0.001). 

### 6.6. Direct and Total Effects of the Path Model 

The results of the direct and total effects in the path model are shown in Table 4. BIS and BAS_drive had significant direct effects (β = 0.147, *p* < 0.05; β = 0.250, *p* < 0.001, satisfied H7) and total effects (β = 0.315; β = 0.250, satisfied H9) on anxiety. BIS and BAS_drive explained 7.3% of anxiety. BIS and BAS_fun seeking had a significant direct effect (β = 0.315, *p* < 0.001, satisfied H11; β = 0.366, *p* < 0.001, satisfied H14) and total effects (β = 0.147; β = 366) on low self-control. BIS and BAS_fun seeking explained 20.3% of low self-control. The variables that showed significant direct effects on SAP were BIS (β = 0.165, *p* < 0.01, satisfied H1), BAS_drive (β = 0.248, *p* < 0.001, satisfied H3), anxiety (β = 0.278, *p* < 0.001, satisfied H5), and low self-control (β = 0.259, *p* < 0.001, satisfied H6). BIS, BAS_drive, anxiety, and low self-control also had significant total effects on SAP, respectively (β = 0.287; β = 0.299; β = 0.278; β = 0.259) and explained 38.4% of SAP.

### 6.7. Indirect Effect of the Path Model

Table 5 suggested the results of the bootstrap test of the mediating effects of the variables. The indirect effects did not include zero in the 95% bootstrap confidence interval, which proved that the indirect effects were significant and the mediating effects existed. Based on the results, the indirect effect on the path of BIS → anxiety → SAP was 0.040 (*p* < 0.05, 95% CI [0.009, 0.081]); indirect effect on the path of BAS_drive→ anxiety → SAP was 0.069 (*p* < 0.001, 95% CI [0.024, 0.132]), the indirect effect on the path of BIS → low self-control → SAP was 0.080 (*p* < 0.001, 95% CI [0.042, 0.131]); and the indirect effect on the path of BAS_fun seeking → low self-control → SAP was 0.093 (*p* < 0.001, 95% CI [0.050, 0.156]); indicating significant mediating roles of anxiety and low self-control.

## 7. Discussion

The current study comprehensively explored the direct and indirect effect of psychological variables on smartphone addiction in the sample of smartphone-based SNS users. Most existing studies on smartphone addiction have been conducted on general smartphone users without considering application contents that they mainly use. Therefore, we strongly believe that the findings will make a unique contribution to the context of existing work.

### 7.1. Direct, Indirect, and Total Effect of BIS, Low Self-Control, Anxiety, and BAS

One of our major findings revealed that BIS had significant direct and total effects on SAP (H1). In other words, BIS was a factor that independently influences addictive smartphone use, which confirms a previous finding e.g., [5]. Highly activated BIS causes the momentary inhibition of ongoing behavior and enhanced vigilance, which are linked with subsequent reward-seeking behaviors to avoid aversive cues [30,45]. Following this behavioral mechanism, individuals with increased BIS sensitivity may quickly respond with negative cues and engage in approach rewarding stimuli as a result of activating avoidance behavior. In this sense, smartphone users with high BIS may use their devices as a way of actively avoiding their daily struggles, which could lead the users to addictive smartphone use. Adults in their 20 to 40, who were the participants of this study, have higher stress levels due to pressures from academic achievement, career, and social relationships than other generations [56]. In order to escape from stressful daily events, adult smartphone-based SNS users with high levels of BIS may indulge pleasurable behaviors, such as using SNS applications via smartphones. Exploring stressful events or negative emotions that activate inhibitory coping strategies would be necessary when establishing intervention and prevention strategies for smartphone-based SNS users’ addictive smartphone use. 

BAS_drive had significant direct and total effect on SAP (H3). This result is in line with previous findings that suggested the sensitivity to drive is positively associated with media addiction properties [57,58]. Smartphones have seductive media contents that provide social interaction, a sense of belonging, and novel information, such as SNS. Given that the persistence to obtain goals (BAS_drive) is closely connected to active pursuit of desired consequences [25,36,58], smartphone-based SNS users with high sensitivity on this BAS dimension may vigorously approach enjoyable smartphone contents, including SNS applications, to satisfy various needs and obtain rewarding outcomes. This might make the users be prone to addictively use their devices and lead them to smartphone addiction. Further intervention should pay attention to decreasing addictive motivations for relentlessly pursuing rewarding goals and outcomes. Furthermore, providing insight on possible negative consequences of the addictive use of pleasurable smartphone content would be needed.

Anxiety also had significant direct and total effect on SAP (H5). This finding was similar to those of literature that found the positive association between addictive smartphone use and high level of anxiety [19,30,31]. As SNS is considered as a way of fulfilling relational needs to stay up to date, to connect with others, and not to miss out [10,59], those who actively use SNS have a strong desire to participate in what others are doing and experience a pervasive apprehension of not being able to engage in social interactions [59,60]. Such anxious thoughts drive individuals to fulfill their needs of social belongingness, thereby leading them to stay connected to social networks [57]. Given that our participants consisted of those who mainly use their smartphones for SNS, they may have a fear of being ostracized from the social networking community. Such a social form of anxiety might lead the users to repeatedly use SNS applications via their smartphones to alleviate their anxious mood and stay connected with others. In turn, their behavior may raise the odds of SAP. Thus, reducing anxiety that underlies social networking activities may promote desirable smartphone usage habits of smartphone-based SNS users.

Low self-control had significant direct and total effect on SAP (H6), which is consistent with prior studies that postulated a positive relationship between a low level of self-control and addictive smartphone use, e.g., [20,39,41,42]. SNS applications are so easily accessible that smartphone users can use those apps only with the slight touch of their fingertips, and this relative immediacy is linked to pleasurable activities [4]. Such a process can make it difficult for users to quit enjoyable smartphone usage behavior. Similarly, smartphone-based SNS users with low self-control may frequently choose to use their devices for using SNS rather than to focus on their duties. Such device usage patterns may lead the users to addictively use their smartphones that ultimately increases the risk of SAP. Hence, enhancing self-control capacity is an effective strategy to prevent addictive smartphone use of individuals whose main smartphone usage is SNS. 

In this study, BIS and BAS_drive had an indirect effect on anxiety that mediated the relationships of BIS–SAP and BAS_drive–SAP. Those two variables also had a direct effect on anxiety (H7, H9). The findings were in line with existing studies identifying a mediating role of anxiety on addictive smartphone use [61,62]. A high level of anxiety is positively related to the activation of BIS as BIS can manifest one’s sensitivity to aversive results [58]. Indeed, individuals with high BIS sensitivity, who are known to have higher anxiety levels [36], tend to become more anxious towards potential threats [22]. Given that the activation of BIS predicted a lack of confidence in social situations that withdraw direct social interaction [62], smartphone-based SNS users with high BIS may tend to frequently focus on negative social cues that induce anxiety. In turn, they might prefer to use indirect communication methods, such as SNS applications, in order to maintain their social relationships without any potential threats. This may explain the association among anxiety, BIS, and SAP of smartphone-based SNS users. Furthermore, underlying goal-directed motivations, which are linked to rewarding experiences, can affect smartphone users’ ability to control their behavior while using smartphone applications. As the use of smartphone applications can be linked with users’ motivation to obtain satisfaction, the users with high BAS_drive may sustain to pursue their long-term goals in the real world. Instead, they may choose to use applications that offer immediate pleasurable feelings, in order to satisfy their goal-directed motivations for using smartphones. Such a smartphone usage behavior may cause negative feelings as the users might think they have not pursued the important long-term goals they need to achieve. Given that individuals facilitate active approach behaviors to reduce anxiety [45], smartphone-based SNS users with high sensitivity on BAS_drive may more vigorously approach enjoyable smartphone contents, such as SNS applications, as a way of alleviating the anxious mood. This process may explain how anxiety can mediate the relationship between BAS_drive and SAP. When establishing intervention strategies for smartphone-based SNS users’ addictive smartphone use, paying attention to anxiety symptoms, the level of behavioral inhibition, and the goal-directed motivations to use smartphones would be needed. 

Moreover, our result revealed that BIS and BAS_fun seeking indirectly affected SAP while mediating the paths of BIS–SAP and BAS_fun seeking–SAP. Those two variables also had a direct effect on low self-control (H11, H14). Similar to a previous study that emphasized the bridging role of self-control on addictive smartphone use [20,63], our results showed that self-control played significant mediating roles in the influence of the behavioral motivation systems on SAP. Smartphone applications are not regarded as the essential function of the device, such as call or text, but as supplementary contents that smartphone users can use based on their free will, purpose, or behavioral motivations. For instance, individuals can use their smartphone applications as a way of releasing stress or pursuing enjoyment. That is, it can be inferred that the frequency of smartphone applications usage may depend on either individuals’ behavioral motivation for using smartphone or their ability to control their voluntary behaviors. Given both the high motivation of avoiding threats and the tendency to seek fun can lower one’s self-control capacity [47], high sensitivity of BIS and BAS_fun seeking may decrease smartphone-based SNS user’ self-control ability, which may induce the failure of controlling their addictive smartphone usage. Therefore, interventions for smartphone-based SNS users require a thorough understanding of the users’ withdrawal motivation and tendency to seek fun activities which influence their self-control capacity, such as what they want to avoid and what they enjoy while using their devices. 

Reward responsiveness from BAS has neither a significant direct nor indirect effect on all study variables in our research. This result is similar to the previous findings that BAS_reward responsiveness was not directly related to addictive smartphone use [64], anxiety [65], and self-control [44], respectively. Perhaps other subcomponents of BAS, drive and fun-seeking, play a more dominant role in explaining smartphone-based SNS users’ underlying motivation of approaching behaviors. However, our result deviated from previous literature that identified the positive relationships between the reward responsiveness and addictive smartphone use [5], anxiety [66], or self-control [67]. Future research should investigate more clear relationships between BAS_reward responsiveness and other psychological variables. 

### 7.2. Limitations

While the contributions of this study are novel and informative, there are few limitations. First, there might be a reporting bias which exists in most smartphone studies which tend to adapt self-report methods as our data consisted of self-report surveys [64]. It would be recommended for future researchers to use interview methods or numerically robust instruments such as applications. Second, although we explained the characteristic of anxiety in the context of social experience in order to explain the direct path of anxiety and SAP, it should be tested in the future study as we did not measure a social form of anxiety. The Fear of Missing Out (FoMO) [65], defined as an anxiety of not being able to engage in social networks, would be an adequate variable for future studies. Third, as we used the path analysis method and thus did not use latent variables in the analysis, there was no measurement model and the measurement error might have not been controlled. In addition, we could not test the common method variance as path analysis does not include the measurement model. Future studies need to conduct the validation of the measurement model of variables in order to enhance the validity of the research. Lastly, the participants in this study were adults living in Korea. Hence, the generalizability of our results might be limited despite the value of the findings. Future studies should include smartphone-based SNS users with more diverse sociodemographic factors (e.g., age, cultural background), as well as taking into account gender differences.

## 8. Conclusions

Despite the abovementioned limitations, the present study provides new insights into the existing literature on addictive smartphone use as there have been no studies that investigated the direct and indirect effect of psychological variables contributing to smartphone addiction, while targeting only smartphone-based SNS users. Our results contain some interesting indications that are useful for clinicians working in the field of smartphone addiction. Exploring inhibitory coping strategies, motivations to relentlessly pursue rewarding goals, and willingness to seek out rewarding experiences would be necessary to establish intervention and prevention strategies for smartphone addiction of smartphone-based SNS users Furthermore, reducing the level of anxiety and enhancing the self-control capacity may prevent addictive smartphone usage of smartphone-based SNS users.

## Figures and Tables

**Table 1:** Participants’ sociodemographic characteristics (*n* = 433).

Variable	Category	*n* (%) or M ± SD
**Sex**	Male	218 (50.3)
	Female	215 (49.7)
**Age**		30.75 (7.84)
**Level of education**	High school graduated	36 (8.3)
	Above university/college graduated	397 (91.7)
**Job status**	Student	102 (23.6)
	Salaried worker	233 (53.7)
	Professional	51 (11.8)
	No job	15 (3.5)
	Others	32 (7.4)
**Relationship/Marriage status**	Not married	267 (61.7)
	Living with a partner	5 (1.2)
	Married	154 (35.6)
	Divorced/Separated	7 (1.6)
**Economic status**	High	51 (11.8)
	Middle	181 (41.8)
	Low	201 (46.4)

M = mean, SD = standard deviation, *n* (%) = number (percentage).

**Table 2:** Smartphone and SNS application use among participants (*n* = 433).

	M (SD)	*n* (%)
**Hours of daily smartphone use**	10.44 (7.73)	
≥10		289 (66.7)
<10		144 (33.3)
**Hours of daily SNS applications via smartphone**	4.61 (4.06)	
≥5		320 (73.9)
<5		113 (26.1)
**Hours of daily SNS use once started using the applications**	1.86 (1.50)	
≥2		344 (79.4)
<2		89 (20.6)

M = mean, (SD) = standard deviation, *n* (%) = number (percentage).

**Table 3:** Correlation coefficients, means, and standard deviations of the study variables (*n* = 433).

Variables	1	2	3	4	5	6	7	M (SD)	Range	Kurtosis	Skewness
	r	r	r	r	r	r	r				
1. BIS	1	0.653 **	0.395 **	0.361 **	0.186 **	0.361 **	0.331 **	18.86 (3.23)	8–28	0.419	0.022
2. BAS_r		1	0.714 **	0.708 **	0.185 **	0.326 **	0.300 **	13.59 (2.78)	5–20	0.934	−0.483
3. BAS_d			1	0.674 **	0.247 **	0.237 **	0.359 **	9.54 (2.14)	4–16	0.569	0.049
4. BAS_f				1	0.165 **	0.369 **	0.258 **	10.25 (2.28)	4–16	0.430	−0.323
5. Anxiety					1	0.467 **	0.479 **	18.43 (8.12)	10–50	1.345	1.219
6. Low self-control						1	0.476 **	36.08 (6.89)	17–58	0.167	−0.108
7. SAP							1	32.41 (7.24)	15–54	−0.300	0.049

** *p* < 0.01. M = mean, (SD) = standard deviation, BIS = behavioral inhibition system, BAS_r = behavioral activation system_reward responsiveness, BAS_d = behavioral activation system_drive, BAS_f = behavioral activation system _fun seeking, SAP = Smartphone Addiction Proneness.

**Table 4:** Standardized direct and total effects of study variables (*n* = 433).

Path		Direct Effect	Total Effect	SMC
		β (*p*)	β	%
**Anxiety**	← BIS	0.147 (0.019)	0.315	7.3
	← BAS_r	−0.099 (0.273)	−0.099	
	← BAS_d	0.250 (<0.001)	0.250	
	← BAS_f	0.014 (0.846)	0.014	
**Low self-control**	← BIS	0.315 (<0.001)	0.147	20.3
	← BAS_r	−0.089 (290)	−0.089	
	← BAS_d	−0.070 (0.282)	−0.070	
	← BAS_f	0.366 (<0.001)	0.366	
**Smartphone addiction proneness**	← BIS	0.165 (0.002)	0.287	38.4
	← BAS_r	−0.126 (0.090)	−0.176	
	← BAS_d	0.248 (<0.001)	0.299	
	← BAS_f	−0.005 (0.935)	0.094	
	← Anxiety	0.278 (<0.001)	0.278	
	← Low self-control	0.259 (<0.001)	0.259	

SMC = square multiple correlation, BIS = behavioral inhibition system, BAS_r = behavioral activation system_reward responsiveness, BAS_d = behavioral activation system_drive, BAS_f = behavioral activation system _fun seeking.

**Table 5:** Standardized indirect effects of study variables (*n* = 433).

Mediator	Dependent Variable ← Independent Variable	Indirect Effect	95% CI	
		β (*p*)	Lower Limit	Upper Limit
**Anxiety**	SAP ← BIS	0.040 (0.011)	0.009	0.081
	SAP ← BAS_r	−0.027 (0.232)	−0.078	0.021
	SAP ← BAS_d	0.069 (<0.001)	0.024	0.132
	SAP ← BAS_f	0.004 (0.826)	−0.033	0.040
**Low self-control**	SAP ← BIS	0.080 (<0.001)	0.042	0.131
	SAP ← BAS_r	−0.023 (0.272)	−0.075	0.018
	SAP ← BAS_d	−0.018 (0.270)	−0.057	0.016
	SAP ← BAS_f	0.093 (≤0.001)	0.050	0.156

CI = confidence interval, BIS = behavioral inhibition system, BAS_r = behavioral activation system_reward responsiveness, BAS_d = behavioral activation system_drive, BAS_f = behavioral activation system _fun seeking, SAP = Smartphone Addiction Proneness.

## Data Availability

Survey material is confidential as it may include unanonymized personal information, but minimal data set can be made available upon request. Contact information: Bohyun Jang, Hankook Research Inc.; bhjang@hrc.co.kr.
